# Assessment of the Hepatitis C Surveillance System in Henan, China: 2014~2016

**DOI:** 10.1155/2018/8942152

**Published:** 2018-09-26

**Authors:** Jie Li, Xuelan Shang, Yan Liang, Wenjie Yang, Zhe Wang, Hezhao Ji

**Affiliations:** ^1^STD and AIDS Prevention Institute, Henan Center for Disease Control and Prevention, Zhengzhou, Henan, China; ^2^AIDS Prevention Section, Erqi District Center for Disease Control and Prevention, Zhengzhou, Henan, China; ^3^National Microbiology Laboratory, Public Health Agency of Canada, Winnipeg, Manitoba, Canada

## Abstract

**Objectives:**

To access the Hepatitis C (HepC) surveillance program in Henan, China, 2014~2016.

**Methods:**

A total of 8,448 HepC-relevant cases were reviewed and this data was then inquired against the 6,147 archived HepC reports during the same time period. The performance of the HepC surveillance program was evaluated using parameters including Timely Reporting Rate (TRR), pairs of Report Sensitivity (RS)/Underreporting Rate (UR), and False Report Rate (FRR)/Predictive Value Positive (PVP). Longitudinal comparisons of report quality over the three examined years were conducted to determine the temporal trend of HepC surveillance accountability.

**Results:**

All HepC reports were submitted within 24 hours post diagnosis, and TRR rates for all examined years remained at 100%. The RS rates significantly improved overtime for clinically diagnosed HepC cases (CDHC) (2014:60.32%, 2015:68.13%, and 2016:82.83%), whereas the RS for confirmed HepC cases (CHC) remained relatively constant (80.77%, 88.64%, and 85.82%). The FRR rates for CDHC and CHC in 2015~2016 were both approximately 30% but at 23.61% and 51.85%, respectively, in 2014.

**Conclusions:**

The HepC surveillance system in Henan remains effective and consistent improvement in report accountability was observed over time. However, some issues concerning especially RS and PVP remained to be addressed for ensuring data accountsbility.

## 1. Introduction

An effective notifiable disease surveillance system is essential in public health management especially in response to hidden communicable disease outbreaks. It plays a key role in identifying new disease endemic/epidemic, monitoring the evolution of disease prevalence, assessing the efficacy of public health interventions, and informing the public health decision making. This has been demonstrated in many disease outbreaks such as SARS in 2002~2003 [1, 2], Ebola in 2014~2016 [3], and Zika infection outbreak in 2016 [4]. In comparison to acute infections, chronic communicable diseases, such as Hepatitis C (HepC), are often less noticeable despite their significant consequences at both individual and population levels. Subsequently, surveillance on such diseases may not attract sufficient attention although it is equally valuable concerning public health.

HepC, caused by Hepatitis C virus (HCV) infection, is now a class B notifiable communicable disease in China [5]. It is one major liver disease contributing to 60~70% of hepatic fibrosis, 5~20% of cirrhosis, and 1~5% of hepatocellular carcinoma [6, 7]. Over the past decade, the number of reported HepC cases has been increasing in China with annual incidence rate reaching 15.09 per 100,000 in 2016 [8]. Remarkably, approximately 1/7 of the newly reported HepC cases in China were from Henan, one of the 34 provincial–level jurisdictions located in central China with a population of 107 million [9]. The HepC prevalence in Henan reached 29.89/100,000 in 2015 nearly doubling the national average and making Henan one of the provinces hit the hardest by HepC in China.

Together with other major provinces in China, the HepC surveillance program started in Henan when HepC was officially included in the national notifiable disease surveillance system in 1950. It started with hardcopy report card and later transitioned to electronic filing in 1985 and then to web-based, real-time case reporting since 2004. As required by the Chinese national standard on notifiable disease reporting, all clinicians responsible for HepC diagnosis and treatment are obligated to report newly diagnosed HepC cases within 24 hours postdiagnosis through the online surveillance system [10]. Duplicate reports are automatically corrected while all newly reported cases being inquired against the existing cases in the HepC surveillance system. The national standard HepC case definition was first published by the National Health and Family Planning Commission of China in 2008 and it is currently the only standard for HepC diagnosis and reporting in China [11]. According to this standard, the HepC case is defined as either Clinical Diagnosed Hepatitis C (CDHC) or Confirmed Hepatitis C (CHC). CDHC refers to those being serapositive for anti-HCV antibodies in the presence of one of the following: (1) potential HCV exposure (i.e., exposure to HCV via HCV contaminated blood or blood products, sexual contacts with HCV infected partner(s), or vertical HCV transmission); (2) HepC-like clinical symptoms; and (3) abnormality of liver functions. CHC is defined when CDHC cases are confirmed positive for HCV-RNA assay suggestive of active HCV replication. All CDHC cases that have been confirmed as CHC are to be updated in the surveillance system by the submitting institutes to avoid double counts. While reporting, all CHC cases are further categorized as acute or chronic HepC. The acute cases refer to those who acquired HCV infection within the past 6 months, while chronic HepC refers to those who have been HCV-infected for >6 months.

As aforementioned, the current HepC reporting system has been implemented since 2004. Report on notifiable communicable diseases via the China Information System for Disease Control and Prevention (CISDCP) is currently the main channel of collecting the disease data in China. The data and analysis results from this system inform government of developing targeted disease prevention and control strategies. The heavy disease burden as well as the public health challenge raised from the high HepC prevalence in Henan necessitates a reassessment of the current HepC surveillance system in the province especially in terms of its accuracy and efficiency. This provides valuable information on the efficacy and accountability of the existing HepC surveillance system and helps to identify areas where further improvement is required. By retrospective analysis of HepC relevant patient records and reported HepC cases from 425 health agencies across Henan province during 2014~2016, we systemically assessed the quality of the reports from the provincial HepC surveillance system in this study. The trends of the HepC surveillance report quality improvement over the three examined years were also explored.

## 2. Methods

### 2.1. Selection of HepC Reporting Institutes for Evaluation

**Figure 1:**
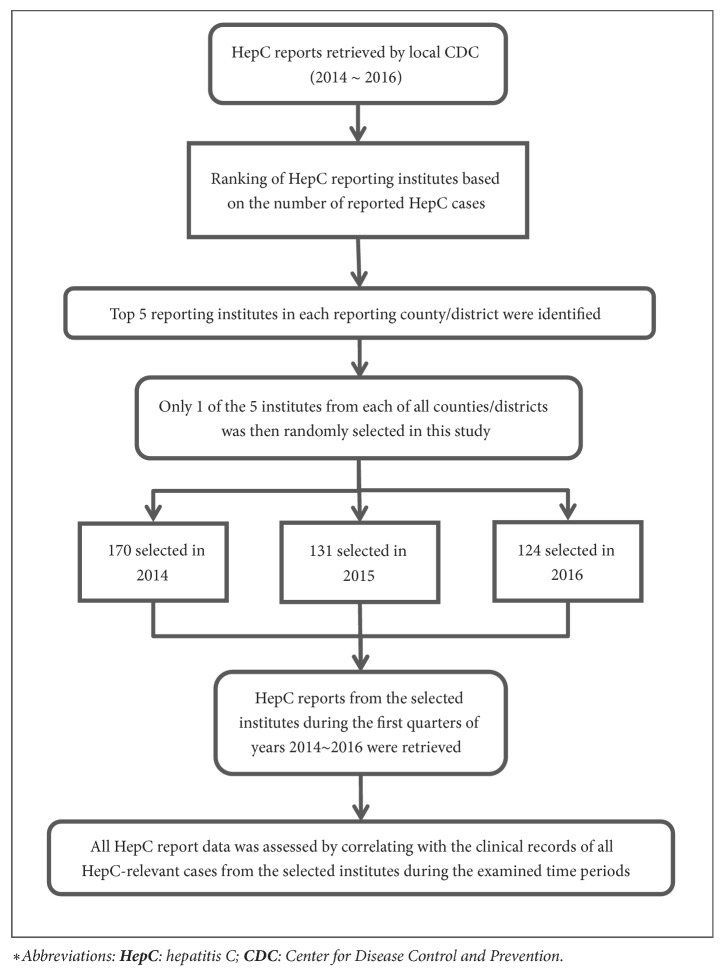
Study design and workflow.

Henan province is located in central China consisting of 18 prefectures/cities and many counties/districts. A total of 1,707 health institutes across Henan province are listed as HepC reporting units that are required to report all newly diagnosed HepC cases through CISDCP. These institutes include 1,521 hospitals, 164 maternity and child care centers, and 22 specialized disease prevention and treatment centers [9]. And HepC reports from these institutes are monitored by 179 local Centers for Disease Control and Prevention (CDC) [12]. To systematically assess the HepC reporting quality, on-site reviewing of all HepC relevant cases was conducted for randomly selected sites in accordance with the Chinese national infectious diseases surveillance guideline and regulations. According to the selection criteria outlined in Figure 1, a total of 425 institutes were selected for the years of 2014~2016 (170 for 2014, 131 for 2015, and 124 for 2016).

### 2.2. HepC Surveillance Report Quality Assessment

HepC reports from each of the selected health institutes during the designated surveillance years were retrieved, followed by onsite visits for a thorough check of all HepC-related cases in the corresponding reporting institutes during the examined time periods. History information of all cases, including the epidemiological data, clinical symptoms, and laboratory testing results (i.e., Anti-HCV antibody, HCV-RNA, liver function test, histopathological examination, CT, MRI, etc.) of HepC relevant patients in these institutions was reviewed. Each HepC related case was name-searched and matched against the records archived in the HepC surveillance system.

The Chinese national standard was used as the gold standard to evaluate the surveillance system [11]. The parameters employed in the quality assessment of the HepC reports from the examined institutes included (1) Timely Reporting Rate (TRR): the proportion of HepC cases reported within the required time frame (⩽ 24 hours post diagnoses); (2) Report Sensitivity (RS): the proportion of HepC cases correctly reported through the surveillance system; (3) Underreporting Rate (UR): the proportion of reportable HepC cases not captured by the surveillance system; (4) False Report Rate (FRR): the proportion of HepC cases falsely reported through the surveillance system; (5) Predictive Value Positive (PVP): the proportion of truly reportable cases among all reported HepC cases.

All data was analyzed using SPSS 19.0 (IBM Corp., Armonk, NY, USA). Cruskal-Wallis test, linear-by-linear association analysis, and Chi-square Test were employed, where applicable, to compare the recorded surveillance data with the clinical information of HepC relevant cases from health institutions through 2014~2016. The evaluation was performed by the professional staff from the local Center Disease Control and Prevention (CDC) System. All data included in this study was double checked by different staff members to ensure its accuracy.

## 3. Results

### 3.1. Retrieved HepC Reports and HepC: Relevant Cases from the Selected Institutes during the Examined Time Periods in 2014~2016

As indicated in Figure 1, 425 HepC reporting institutes in Henan province were selected from years 2014~2016 for this study. A total of 6,147 HepC cases were reported by these institutes through the HepC surveillance system during this time period. In contrast, the case-by-case reviewing of the HepC-relevant cases revealed that 8,448 HepC-related cases presented themselves in these institutes during this period. The sociodemographic information for all the reported and onsite reviewed HepC cases is shown in Table 1.

### 3.2. Assessment of the Quality and Accountability of HepC Surveillance Reports

All 6,147 reported HepC cases were recorded in the surveillance system within 24 hours postdiagnosis and, therefore, the TRR rate remained at 100.00% for all three examined years. The median reporting time durations (in hours) between HepC diagnosis and reporting varied at 4.30 (0.05~23.92) in 2014, 3.17 (0.15~23.83) in 2015, and 3.38 (0.13~23.77) in 2016. Cruskal-Wallis test showed the reporting times varied among the three examined years while statistical differences were observed between year 2015 and the two other compared years (p<0.05).

Despite the high TRR rates, the results from other assessment indices were less optimal. RS and UR rates reflect the percentages of reportable cases that have been reported (RS) or underreported (UR), respectively. They evaluate the data completeness of the HepC reports in the surveillance system. The RS rates increased continuously over the three examined years from 67.50% in 2014 to 84.18% in 2016 (p<0.001). Correspondingly, the UR rates dropped from 32.50% in 2014 to 15.82% in 2016 (p<0.001) (Table 2). When the cases from the CDHC and CHC categories were analyzed separately, high RS rates (>80%) were observed consistently for CHC cases and significantly higher RS rates were observed in the years of 2015 and 2016 as compared to that of 2014 (p<0.01). However, the RS rate for CDHC cases was as low as 60.32% in 2014, but it increased significantly in the two following years (p<0.001), reaching 82.83% in 2016. (Table 2)

The PVP and FRR rates measure the accuracy of the HepC reports by calculating the percentile of accurately (PVP) or falsely reported (FRR) cases in the surveillance system. Continuous improvements were observed in HepC report accuracy as indicated by the PVP values for years 2014, 2015, and 2016 (at 61.29%, 69.48%, and 71.41%, p<0.001). When CDHC and CHC cases were reviewed separately, the PVP rates for both categories remained at approximately 70% for 2015 and 2016, although 2014 showed a different scenario (PVP values for CDHC and CHC at 76.39% and 48.15% respectively), suggesting some unexpected issues might have occurred.

### 3.3. Characterization of Falsely-Reported HepC Cases from the Examined Reporting Institutes during 2014~2016

False reports contribute to a significant portion of the inaccuracy of HepC reporting. Although consistent FRR reduction was observed from 2014 to 2016, FRR remains to be the major issue in HepC surveillance system. For instance, an FRR of 28.59% was observed even in 2016 (Table 2) suggesting that nearly 30% of the reported HepC cases were not reported accurately. To determine the factors contributing to the high FRR rates, we further characterized the falsely reported HepC cases. As indicated in Table 3, the main type of reporting errors is misclassification of CDHC and CHC cases. This was especially the case for CDHC cases, of which many were reported as CHC, although significant improvement was observed over the examined period. In contrast, misclassification of CHC cases was an issue of less significance. Besides the misclassification errors, another type of error resulting in high FRR was false reporting on cases that required no report. Such cases included those who are (1) HCV serapositive and HCV-RNA negative in the absence of any clinical manifestations or liver functional abnormality, suggestive of past HCV infection only; (2) HCV serapositive with no HCV-RNA assay results and no recognizable exposure history or clinical symptoms or liver functional abnormality, suggestive of uncertain HepC status.

## 4. Discussion

Surveillance on notifiable communicable diseases through the CISDCP has been routinized in China and it has become a key data source that informs public health decision-making for targeted disease prevention and control. Without any doubt, the data validity and quality from such surveillance system is vital for ensured accountability. Understandably, this surveillance system involves both clinical and public health institutes with professionals of different expertise. Errors or biases at any node of this comprehensive workflow may impair the quality of eventual data output. To our knowledge, this is the first study systematically evaluating the HepC surveillance program in Henan province using data from 3 consecutive years. It not only evaluates the quality and accuracy of the HepC surveillance reports in the examined years but also inspects the trends of HepC surveillance improvement over time.

Timely reporting is essential for surveillance on any notifiable disease such as HepC. CISDCP requires all class B notifiable diseases being reported within 24 hours postdiagnosis. Our data showed that the TRR rates remained at 100% for all three examined years indicating that all HepC reports were submitted within the mandatory timeframe. This suggests that the current HepC surveillance system has been well accepted and is being executed effectively by all the participating health institutes. Although HepC cases were reported in a timely manner, our study did identify some key issues that remain to be addressed to further enhance HepC surveillance practice.

RS and UR reflect the portion of all reportable HepC cases being reported or underreported in the system. A high RS rate or low UR rate ensures the completeness and integrity of the report data in reflecting the true HepC incidence and prevalence in the region. We observed that the RS rates for HepC kept increasing from 2014 to 2016 reaching 84.18% in 2016, suggestive of the continued improvements of the HepC surveillance. However, it is also noteworthy that even in 2016 the UR rate was 15.82%, indicating that nearly 1 out of 6 reportable HepC cases was not captured by the surveillance system. Although these rates are comparable to the other alike disease surveillance programs in China [13–15], they highlight the need for further improvements in this regard. This issue was of more concern for CDHC cases as compared to CHC, suggesting that a clearer definition and diagnosis standard for CDHC is urgently required. In a separate analysis, we also observed consistently lower RS rates for acute HepC cases as compared to those for chronic HepC in CHC category (*data not shown*). This is in concordance with observations from two other studies completed in 2014 and 2016 in China [16, 17]. Both of these observations suggest that these trends are rather systematic and relevant to some fundamental issues. For instance, a clearer definition and more workable diagnosis criteria are urgently required for HepC.

PVP and FRR measure the portion of true and falsely reported cases in all HepC reports. These two parameters measure the report accuracy of a surveillance program. Understandably, a low PVP or high FRR rate significantly impairs the surveillance data accountability since it may mislead subsequent HepC management strategies. In concordance with the results from RS/UR analysis, our PVP/FRR results further confirmed that the accuracy of HepC surveillance report was improving over the three examined years, reaching 71.41% (for PVP) and 28.59% (for FRR), respectively, in 2016. The report accuracy was slowly improving but still rather alarming from public health perspectives since nearly 29% of all reported cases were recorded by error. We have demonstrated clearly here that misclassification of CDHC/CHC and false reporting on cases who were solely HCV serapositive with no indication of active HCV infection. Approximately 30% of HCV infected cases may spontaneously clear the infection without treatment [7]. These individuals are no longer active HepC cases and require no reporting although they remain positive for HCV antibodies. As for patients from difference categories, no significant PVP/FRR rate difference was observed for CDHC and CHC cases in years 2015 and 2016. However, the PVP rates for CHC were significantly lower than that for CDHC cases (48.15% vs 76.39%). Further investigation revealed that more training on the identification of acute and chronic cases happened in 2015 and 2016.

This study provides an overview of the validity and quality of the current HepC surveillance program in Henan, the heaviest-hit province by HepC in China. Our data confirmed the continuous improvements in the HepC surveillance over time largely contributable to the full appreciation of the importance of the HepC surveillance as well as enhanced technical training among the front-line health professionals. However, the low RS and PVP rates highlight that more issues remain to be addressed to ensure the accountability of the HepC surveillance outcome. Notably, more workable reporting strategies for CDHC cases might be required. Our study indicates that false report on CDHC cases contributes the most to the high FRR due to either being misclassified as CHC or being serapositive only and needless to be reported. Although confirmative HCV RNA assay is recommended for all CDHC cases to rule out those that require no report, it is unrealistic to expect such assay being conducted on all CDHC cases due to the patients' financial constraints and/or limited accessibility to such assays in rural areas [18]. Alternatively, a more operable CDHC case definition might be a better solution to address this issue. The current CDHC definition is rather vague relying mainly on HCV serology testing. A refined CDHC definition with more stringent requirement for HCV-relevant clinical manifestations and concurrent liver function abnormality will help to better define truly reportable HepC cases for surveillance purpose.

On the other hand, we did notice significant improvement on the accuracy and completeness of HepC in the later years of the examined time period. Further investigation suggests that enhanced training of HepC reporting professionals played important role in these progresses. The HepC reporting institutes involve hospitals and medical centers at different levels. It was reported that approximately 50% of the medical professionals were not confirmative of HepC case definitions and reporting standards in China [19, 20]. This necessitates training of all individuals involving in HepC surveillance for ensured data quality.

## 5. Conclusion

We have assessed the performance of HepC surveillance system in Henan, China, during 2014~2016 regarding the reporting timelines, accuracy, and completeness. While continuous improvements were observed during the examined time period, some key issues that impair the HepC surveillance accountability were identified and potential mitigating strategies were discussed here. This information will be applicable for enhanced surveillance programs on infectious diseases such as HepC.

## Figures and Tables

**Table 1:** Sociodemographic characters of all examined Hepatitis C cases.

Variable	2014		2015		2016	
	Reported^Δ^	Assessed^▲^	Reported^Δ^	Assessed^▲^	Reported^Δ^	Assessed^▲^
**Reporting Institutes**						
Hospitals	2067	2598	2029	2639	1602	2733
MCCC^*∗*^	11	11	15	15	9	9
SPTC ^#^	140	169	134	134	140	140

**Age** (yrs)						
0~	21	28	4	9	8	14
15~	90	121	124	146	74	122
30~	381	453	324	401	187	297
45~	916	1131	909	1158	726	1189
60~	810	1045	817	1074	756	1260

**Gender**						
Female	1167	1468	1091	1401	857	1457
Male	1051	1310	1087	1387	894	1425

**Total**	**2218**	**2778**	**2178**	**2788**	**1751**	**2882**

*Notes.* ^Δ^Number of cases recorded in the Hepatitis C surveillance system; ^▲^number of all Hepatitis C cases reviewed during on-site visits; ^*∗*^**MCCC:** maternity and child care centers; ^#^**SPTC:** specialized prevention and treatment centers.

**Table 2:** Assessment of Hepatitis C surveillance system in Henan, China: 2014-2016.

Attributes	2014			2015			2016			P values		
	**OVERALL**	CDHC	CHC	**OVERALL**	CDHC	CHC	**OVERALL**	CDHC	CHC	**OVERALL**	CDHC	CHC
Correctly reported cases (A)	**1300**	754	546	**1496**	560	936	**1224**	661	563	—	—	—
Falsely-reported cases (B)	**821**	233	588	**657**	270	387	**490**	266	224	—	—	—
Underreported-cases (C)	**626**	496	130	**382**	262	120	**230**	137	93	—	—	—

RS=A/(A+C)	**67.50**	60.32	80.77	**79.66**	68.13	88.64	**84.18**	82.83	85.82	<0.001	<0.001	<0.01
UR=C/ (A+C)	**32.50**	39.68	19.23	**20.34**	31.87	11.36	**15.82**	17.17	14.18	<0.001	<0.001	<0.01
PVP=A/(A+B)	**61.29**	76.39	48.15	**69.48**	67.47	70.75	**71.41**	71.31	71.54	<0.001	>0.01	<0.001
FRR=B/ (A+B)	**38.71**	23.61	51.85	**30.52**	32.53	29.25	**28.59**	28.69	28.46	<0.001	>0.01	<0.001

*Notes.* **RS:** report sensitivity, **UR:** underreporting rate, **PVP:** predictive value positive, **FRR:** false report rate, **CDHC:** clinical diagnosed Hepatitis C case, **CHC:** confirmed Hepatitis C case.

**Table 3:** Characterization of falsely reported Hepatitis C cases in Henan, China: 2014~2016.

Year	Report type of diagnosis in network report	Actual type of diagnosis			
		CDHC	CHC	Needless to Report	Subtotal
2014	CDHC	-	123	110	233
	CHC	452	-	136	588
2015	CDHC	-	120	150	270
	CHC	246	-	141	387
2016	CDHC	-	88	178	266
	CHC	126	-	98	224

*Notes.* **CDHC:** clinical diagnosed Hepatitis C case; **CHC:** confirmed Hepatitis C case.

## Data Availability

The data used to support the findings of this study are available from the corresponding author upon request.
